# A Mechanism by which Ergosterol Inhibits the Promotion of Bladder Carcinogenesis in Rats

**DOI:** 10.3390/biomedicines8070180

**Published:** 2020-06-27

**Authors:** Nobutomo Ikarashi, Motohiro Hoshino, Tetsuya Ono, Takahiro Toda, Yasuharu Yazawa, Kiyoshi Sugiyama

**Affiliations:** 1 Department of Biomolecular Pharmacology, Hoshi University, 2-4-41 Ebara, Shinagawa-ku, Tokyo 142-8501, Japan; 2 Department of Clinical Pharmacokinetics, Hoshi University, 2-4-41 Ebara, Shinagawa-ku, Tokyo 142-8501, Japan; hsnmt777-star@yahoo.co.jp (M.H.); onote20218@gmail.com (T.O.); 3 Division of Pharmacology, Faculty of Pharmaceutical Sciences, Teikyo Heisei University, 4-21-2 Nakano, Nakano-ku, Tokyo 164-8530, Japan; t.toda@thu.ac.jp; 4 Department of Clinical Pharmaceutics, School of Pharmaceutical Sciences, University of Shizuoka, 52-1 Yada, Suruga-ku, Shizuoka 422-8526, Japan; yazawa@u-shizuoka-ken.ac.jp; 5 Department of Functional Molecular Kinetics, Hoshi University, 2-4-41 Ebara, Shinagawa-ku, Tokyo 142-8501, Japan

**Keywords:** ergosterol, cyclin D1, androgen receptor, 5α-reductase, bladder cancer

## Abstract

We previously showed that ergosterol has an inhibitory effect on bladder carcinogenesis. In this study, we aimed to elucidate the molecular mechanism by which ergosterol inhibits bladder carcinogenesis using a rat model of N-butyl-N-(4-hydroxybutyl)nitrosamine-induced bladder cancer. The messenger ribonucleic acid (mRNA) expression level of the cell cycle-related gene cyclin D1 and inflammation-related gene cyclooxygenase-2 in bladder epithelial cells was significantly increased in the carcinogenesis group compared with the control group. In contrast, in ergosterol-treated rats, these increases were significantly suppressed. Ergosterol did not affect the plasma testosterone concentration or the binding of dihydrotestosterone to androgen receptor (AR). The mRNA expression levels of 5α-reductase type 2 and AR were higher in the carcinogenesis group than in the control group but were significantly decreased by ergosterol administration. These results suggest that ergosterol inhibits bladder carcinogenesis by modulating various aspects of the cell cycle, inflammation-related signaling, and androgen signaling. Future clinical application of the preventive effect of ergosterol on bladder carcinogenesis is expected.

## 1. Introduction

Bladder cancer is one of the most common urological tumors. It mainly affects men, with an incidence approximately 10 times higher in men than in women [1]. Approximately 70% of bladder cancers are non-muscle-invasive, and transurethral resection (TUR) is a common treatment. Although the prognosis after TUR is good and the 5-year survival rate is at least 95%, the high recurrence rate is a clinical problem [2]. Treatment with anticancer agents [3,4] and bacillus Calmette-Guérin (BCG) infusion [5,6] into the bladder are performed after TUR. However, these treatments impose both a heavy burden on the patient and a large economic burden [7]. Therefore, a new preventive method that can solve these problems is strongly desired.

We previously screened traditional Kampo medicines via a short-term carcinogenicity test to identify preventive agents for superficial bladder cancer. Our results clarified that Choreito strongly inhibits the promotion of bladder carcinogenesis [8]. Choreito is composed of five crude drugs: *Polyporus sclerotium*, *Alisma rhizome*, *Poria sclerotium*, *Donkey glue*, and aluminum silicate hydrate with silicon dioxide. We clarified that among these components, *Polyporus sclerotium* has the strongest inhibitory effect on bladder carcinogenesis [9] and that the ergosterol contained in *Polyporus sclerotium* is the main active ingredient [10]. Furthermore, a long-term carcinogenicity test showed that when ergosterol was orally administered to rats in a model of bladder cancer for 25 weeks, the incidence of bladder tumors was decreased. In addition, experiments using castrated rats revealed that the action of ergosterol may be due to the suppression of androgen signaling by the active metabolite brassicasterol [11]. In this study, we aimed to elucidate the detailed molecular mechanism by which ergosterol inhibits bladder carcinogenesis. In brief, we focused on genes whose expression fluctuates from the early stage of bladder carcinogenesis and investigated the effect of ergosterol on the expression of these genes. In addition, we evaluated the effect of ergosterol on androgen, a hormone that promotes bladder carcinogenesis.

## 2. Experimental Section

### 2.1. Materials

Ergosterol and sodium saccharin (SS) were purchased from Wako Pure Chemicals (Osaka, Japan). *N*-butyl-*N*-(4-hydroxybutyl)nitrosamine (BHBN) was purchased from Tokyo Chemical Industry Co., Ltd. (Tokyo, Japan). Concanavalin A (Con A), α-methylmannoside (α-MM), and dextran-coated charcoal (DCC) were purchased from Sigma-Aldrich Corp. (St. Louis, MO, USA). An RNeasy Mini Kit was purchased from Qiagen (Valencia, CA, USA). A high-capacity complementary deoxyribonucleic acid (cDNA) synthesis kit was purchased from Applied Biosystems (Foster City, CA, USA). A testosterone enzyme immunoassay (EIA) kit was purchased from Cayman Chemical (Ann Arbor, MI, USA). The [1,2,4,5,6,7-^3^H] 5α-dihydrotestosterone ([^3^H]-DHT; 121 Ci/mmol) was purchased from GE Healthcare Bio-Sciences Corp. (Pittsburgh, PA, USA).

### 2.2. Animals

Male 5-week-old Wistar rats were obtained from Japan SLC, Inc. (Shizuoka, Japan). The care and handling of the animals were in accordance with the Hoshi University (approval no. 08-111).

### 2.3. Short-Term Carcinogenicity Study

**Figure 1:**
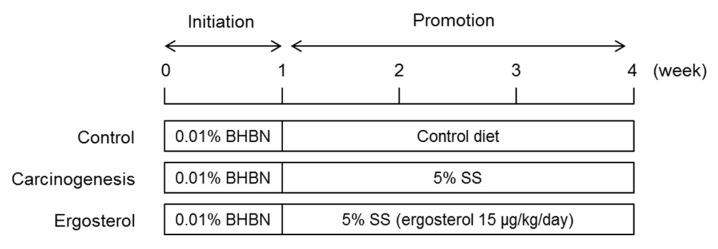
Experimental design.

A short-term carcinogenicity study was performed according to a previous method (Figure 1) [10,12]. In brief, rats were given an aqueous solution of 0.01% BHBN ad libitum for one week as the initiator. For the next three weeks, the rats in the control group were fed a normal diet alone, and those in the carcinogenesis and ergosterol-treated groups were fed a diet containing 5% SS as the promoter. Ergosterol (15 μg/kg/day) was administered orally once daily for three weeks to the rats in the ergosterol-treated group. The rats in the control and carcinogenesis groups received purified water containing 0.18% Tween 80. After the treatment, the rats were anesthetized, after which a blood sample was collected and the bladder was removed.

### 2.4. Measurement of Plasma Testosterone Concentration

Blood samples were centrifuged (1000× *g* for 15 min at 4 °C), and the plasma was stored at −80 °C until the assays were performed. Free testosterone was obtained by centrifugation (12,000 × *g* for 90 s at 37 °C) using an ultrafiltration device (Ultracel YM-30, Millipore Corporation, Bedford, MA, USA). The plasma concentrations of total and free testosterone were enzymatically quantified using the Testosterone EIA kit.

### 2.5. Con A Agglutination Assay

A Con A agglutination assay was performed as described previously with slight modifications [13,14]. In brief, isolated cells from the removed bladders were collected by centrifugation. The cell suspension was mixed with Con A, with or without α-MM. Cell aggregates were counted with a hemocytometer.

### 2.6. Real-Time PCR

The expression levels of target genes were quantified using real-time RT-PCR analysis. Epithelial cells were isolated from rat bladders by scraping, and RNA was extracted with the RNeasy Mini Kit. The total RNA was reverse transcribed with high-capacity cDNA synthesis kit. The forward and reverse primers for target genes are listed in Table 1. The conditions for PCR were denaturation at 95 °C for 15 s, annealing at 56 °C for 30 s, and elongation at 72 °C for 30 s. The messenger ribonucleic acid (mRNA) expression levels were normalized to 18S ribosomal Ribonucleic acid (rRNA) expression levels.

### 2.7. Androgen Receptor (AR) Binding Assay

Maltose binding protein-fused human AR ligand-binding domain (MBP-hAR-LBD; Toyobo Co., Ltd., Tokyo, Japan), [^3^H]-DHT, and ergosterol or DHT were incubated at room temperature for 1 h. After the addition of DCC solution and reaction on ice for 10 min, DCC was removed by centrifugation. The supernatant was added to a liquid scintillation cocktail (Ultima Gold MV, PerkinElmer, Waltham, MA, USA), and the radioactivity of [^3^H]-DHT was then measured in a liquid scintillation counter (TRI-CARB 3100TR, PerkinElmer).

### 2.8. Statistical Analysis

All quantitative results are presented as the means ± standard deviations (SDs). Means were compared using analysis of variance (ANOVA) with correction for multiple comparisons using Tukey’s test for multiple comparisons. A *p* value of <0.05 was considered the level of significance.

## 3. Results

### 3.1. Inhibitory Effect of Ergosterol on Bladder Carcinogenesis

The inhibitory effect of ergosterol on bladder carcinogenesis was examined in a short-term carcinogenicity study using SS as the promoter [10,12] and evaluated in a Con A agglutination assay.

The number of Con A-dependent aggregates was significantly higher in the carcinogenic group than that in the control group. In contrast, the number of cell aggregates was significantly lower in rats treated with ergosterol compared to the carcinogenesis group (Table 2).

From the above results, it was confirmed that ergosterol exhibits inhibitory effect against bladder carcinogenesis, as in the previous reports [10,11].

### 3.2. The mRNA Expression Level of Cyclin D1 in Bladder Epithelial Cells

During bladder carcinogenesis, the proliferation of epithelial cells is accelerated due to cell cycle dysregulation. Cyclin D1 is an important gene involved in cell cycle progression from G1 to S phase, and overexpression of cyclin D1 has been observed in various cancers, including bladder cancer [15,16]. Therefore, we investigated whether the inhibitory effect of ergosterol on bladder carcinogenesis is caused by a change in cyclin D1 expression.

**Figure 2:**
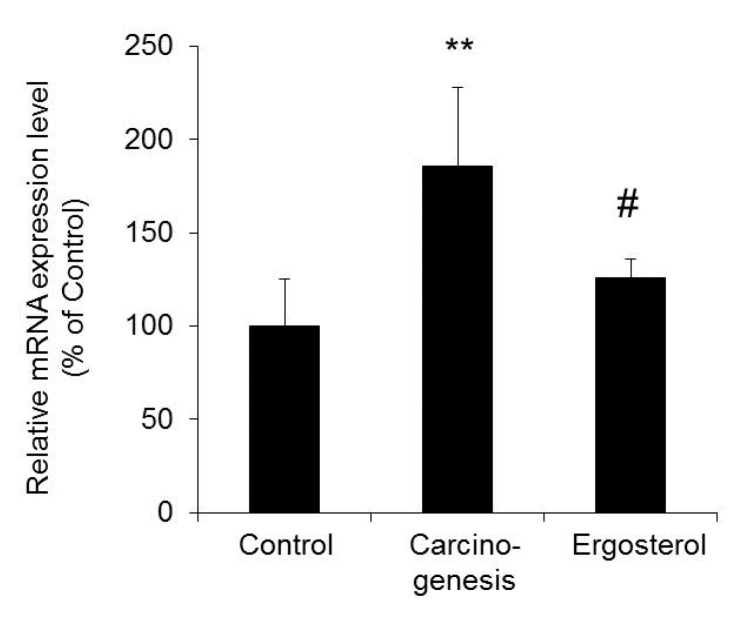
The mRNA expression level of cyclin D1 in bladder epithelial cells. The bladders were removed from rats in the control, carcinogenesis, and ergosterol-treated groups, and the mRNA expression level of cyclin D1 was measured using real-time RT-PCR and normalized to those of 18S rRNA. The data are presented as percentages of the mean value in the control group, which was set at 100%. The data show the mean ± SD from five rats per group. Tukey’s test, ** *p* < 0.01 vs. the control group, # *p* < 0.05 vs. the carcinogenesis group.

The mRNA expression level of cyclin D1 in bladder epithelial cells in the carcinogenesis group was significantly increased by approximately two-fold compared with that in the control group. In contrast, cyclin D1 expression in the ergosterol-treated group was significantly lower than that in the carcinogenesis group and was almost the same as that in the control group (Figure 2).

The above results suggest that the inhibition of bladder carcinogenesis by ergosterol may be due to decreased expression of cyclin D1.

### 3.3. The mRNA Expression Levels of Cyclooxygenase-1 (COX-1) and Cyclooxygenase-2 (COX-2) in Bladder Epithelial Cells

Inflammation in the bladder is considered to be one of the factors responsible for bladder cancer [17]. In general, when inflammation is induced, the expression of COX-2, the rate-limiting enzyme in prostaglandin E_2_ (PGE_2_) production, increases. Although COX-1 expression was not observed in bladder epithelial cells of rats in bladder cancer models, COX-2 expression was increased, which is considered a factor in bladder carcinogenesis [18,19]. Therefore, we investigated whether the change in COX-2 expression is involved in the inhibitory effect of ergosterol on bladder carcinogenesis.

**Figure 3:**
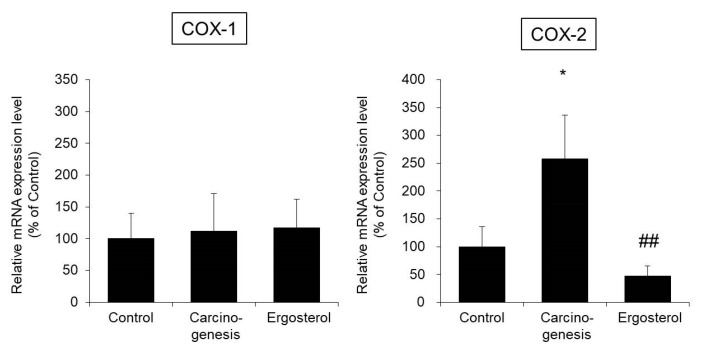
The mRNA expression levels of cyclooxygenase-1 (COX-1) and cyclooxygenase-2 (COX-2) in bladder epithelial cells. The bladders were removed from rats in the control, carcinogenesis, and ergosterol-treated groups, and the mRNA expression levels of COX-1 and COX-2 were measured using real-time RT-PCR and normalized to those of 18S rRNA. The data are presented as percentages of the mean value in the control group, which was set at 100%. The data show the mean ± SD from five rats per group. Tukey’s test, * *p* < 0.05 vs. the control group, ## *p* < 0.01 vs. the carcinogenesis group.

The mRNA expression level of COX-1 in bladder epithelial cells in the carcinogenesis group was almost the same as that in the control group. In addition, no changes in COX-1 expression due to ergosterol administration were observed. On the other hand, the expression level of COX-2 in the carcinogenesis group was significantly increased by approximately two-fold compared with that in the control group. In contrast, the expression level of COX-2 in the ergosterol-treated group was significantly lower than that in the carcinogenesis group (Figure 3).

The above results suggest that ergosterol decreases the expression of COX-2 and suppresses the induction of inflammation.

### 3.4. Effect of Ergosterol on the Plasma Testosterone Concentration

Androgens have been reported to be involved in promoting bladder carcinogenesis [20]. Therefore, we investigated whether the plasma concentration of testosterone was changed by the administration of ergosterol.

**Figure 4:**
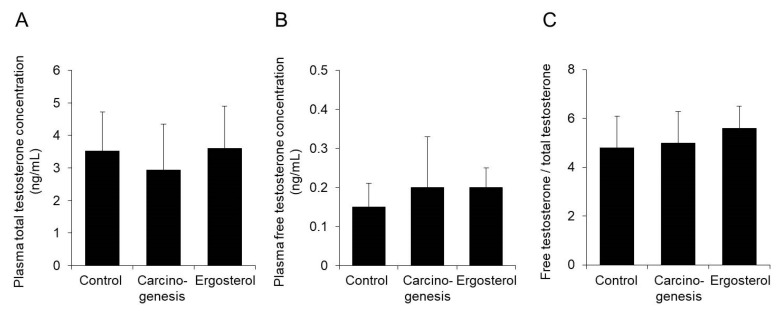
Effect of ergosterol on the plasma testosterone concentration. Plasma was obtained from rats in the control, carcinogenesis, and ergosterol-treated groups. The concentrations of total testosterone (**A**) and free testosterone (**B**) were measured. The ratio of the free testosterone concentration to the total plasma testosterone concentration was calculated (**C**). The data show the mean ± SD from five rats per group.

In ergosterol-treated rats with BHBN-induced bladder cancer, the plasma concentrations of total and free testosterone were almost the same as those in the carcinogenesis group (Figure 4A,B). In addition, the ratio of the free testosterone concentration to the total testosterone concentration was not changed by ergosterol administration (Figure 4C).

The above results indicate that ergosterol is unlikely to suppress androgen signaling by affecting the blood testosterone concentration.

### 3.5. The mRNA Expression Levels of 5α-Reductase and AR in Bladder Epithelial Cells

After uptake by target tissues, testosterone is metabolized to DHT by 5α-reductase. DHT exerts an androgenic effect by binding to AR [20,21]. Therefore, we investigated whether ergosterol alters the expression levels of 5α-reductase and AR in bladder epithelial cells.

**Figure 5:**
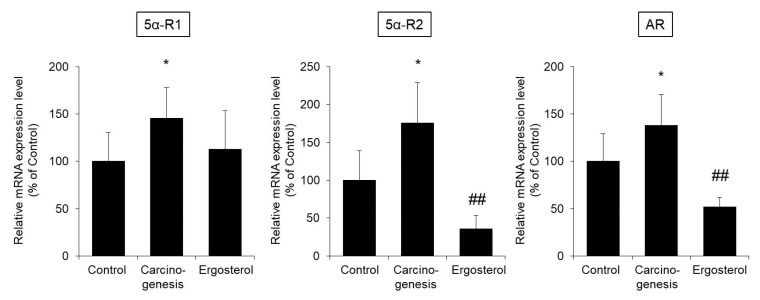
The mRNA expression levels of 5α-reductase and androgen receptor (AR) in bladder epithelial cells. The bladders were removed from rats in the control, carcinogenesis, and ergosterol-treated groups, and the mRNA expression levels of 5α-reductase type 1 (5α-R1), 5α-reductase type 2 (5α-R2), and AR were measured using real-time RT-PCR and normalized to those of 18S rRNA. The data are presented as percentages of the mean value in the control group, which was set at 100%. The data show the mean ± SD from five rats per group. Tukey’s test, * *p* < 0.05 vs. the control group, ## *p* < 0.01 vs. the carcinogenesis group.

The mRNA expression level of 5α-reductase type 1 (5α-R1) in bladder epithelial cells was significantly higher in the carcinogenesis group than in the control group. The expression level of 5α-R1 in the ergosterol-treated group showed a trend of being lower than that in the carcinogenesis group. The expression levels of both 5α-reductase type 2 (5α-R2) and AR were significantly higher in the carcinogenesis group than in the control group. Treatment with ergosterol significantly suppressed these increases in expression (Figure 5).

The above results suggest that ergosterol may inhibit bladder carcinogenesis by decreasing the expression of 5α-reductase and AR and suppressing androgen signaling.

### 3.6. Effect of Ergosterol on the Binding Properties of AR and DHT

We investigated whether ergosterol competitively inhibits the binding of AR and DHT. Specifically, ergosterol was added in the presence of AR and [^3^H]-DHT, and the extent of [^3^H]-DHT exclusion from AR was evaluated.

**Figure 6:**
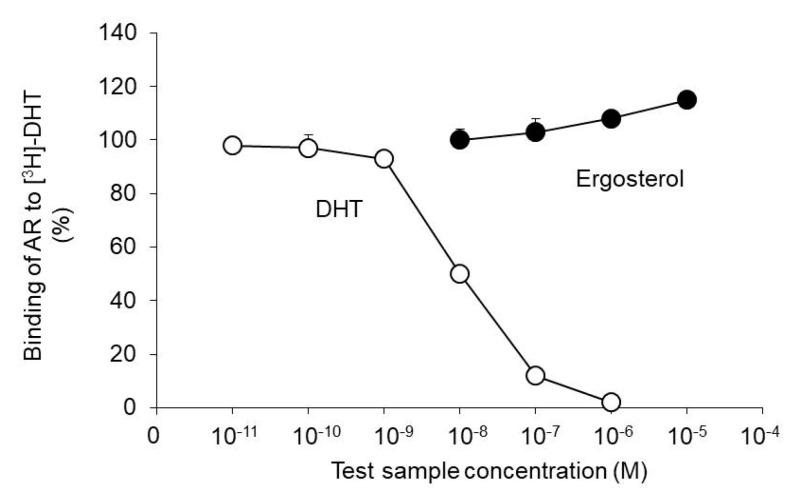
Effect of ergosterol on the binding properties of AR and dihydrotestosterone (DHT). Maltose binding protein-fused human AR ligand-binding domain (MBP-hAR-LBD) and [1,2,4,5,6,7-3H] 5α-dihydrotestosterone ([3H]-DHT) were incubated with ergosterol or DHT. The radioactivity of [^3^H]-DHT bound to AR was measured using a liquid scintillation counter. The data show the mean ± SD from five experiments.

DHT, used as the positive control, showed a concentration-dependent exclusion effect of [^3^H]-DHT from AR. On the other hand, this exclusion effect was not observed with ergosterol (Figure 6).

The above results indicate that ergosterol is unlikely to inhibit the binding of AR and DHT to suppress androgen signaling.

## 4. Discussion

We previously demonstrated that ergosterol, a component of the traditional Kampo medicine Choreito, strongly inhibits the promotion of bladder carcinogenesis [10] and that long-term administration of ergosterol reduces the incidence of carcinogenesis [11]. In this study, we aimed to elucidate the mechanism by which ergosterol inhibits bladder carcinogenesis.

Oral administration of ergosterol to rats in the BHBN-induced bladder carcinogenesis model reduced the Con A-induced cell aggregation rate by 90% (Table 2). This result is similar to that described in a previous report [10,11] and confirms that ergosterol exhibits inhibitory activity against bladder carcinogenesis. Therefore, we conducted a mechanistic analysis in this rat model.

During carcinogenesis, the cell cycle is dysregulated and cell proliferation is accelerated. Cyclin D1 is an important cell cycle mediator, and its expression has been reported to be increased in various cancers, including bladder cancer [15,16]. The expression level of cyclin D1 in bladder epithelial cells was significantly increased in the carcinogenesis group compared with the control group (Figure 2). In the rat model used in this study, carcinogenesis is at an earlier stage than in other rat models of bladder cancer in which increased expression of cyclin D1 has been reported [22,23]. Therefore, cyclin D1 may be one of the important genes whose expression is altered very early in bladder carcinogenesis. Ergosterol was found to suppress the increase in cyclin D1 expression observed in the carcinogenesis group, reducing cyclin D1 expression to a level similar to that in the control group (Figure 2). The above results suggest that suppression of cyclin D1 expression might be one of the mechanisms by which ergosterol inhibits bladder carcinogenesis.

Inflammation is one of the factors that induces the promotion of cell proliferation [17]. The enzyme COX-2 exhibits increased expression during inflammation and carcinogenesis and produces PGE_2_, which has a promotive effect on cell proliferation [24,25]. COX-2 expression has been reported to be increased in bladder epithelial cells during bladder carcinogenesis in rats [18,19] and humans [26,27] and that PGE_2_ has been reported to be involved in the development of bladder cancer [28]. The expression level of COX-2 in the carcinogenesis group was significantly increased compared to that in the control group, and ergosterol suppressed this increase (Figure 3). The above findings suggest that suppression of COX-2 expression might be one of the mechanisms by which ergosterol inhibits bladder carcinogenesis.

Why does ergosterol suppress cyclin D1 and COX-2 expression? Activation of nuclear factor-kappa B (NF-κB) is involved in mediating the increases in cyclin D1 and COX-2 expression during carcinogenesis [29,30], and ergosterol has been reported to suppress NF-κB activation [31,32]. Therefore, ergosterol may reduce the increase in cyclin D1 and COX-2 expression by suppressing the activation of NF-κB. In the future, it will be necessary to investigate the protein expression levels of these genes by Western blotting and immunohistochemistry and to clarify the inhibitory action of ergosterol on NF-κB activity and its mechanism.

Testosterone is metabolized to the active metabolite DHT by 5α-reductase and then binds to AR and exerts an androgenic effect. Flutamide, which has antiandrogenic activity, dose-dependently decreases the incidence of carcinogenesis in the rat model of BHBN-induced bladder carcinogenesis [33]. In addition, the progression of bladder carcinogenesis is suppressed in AR knockout mice [34]. We, therefore, investigated the effect of ergosterol on androgen signaling, which is important for bladder carcinogenesis. Our findings indicated that ergosterol does not affect the blood concentration of testosterone (Figure 4) or the binding of DHT to AR (Figure 6). On the other hand, ergosterol was found to decrease the expression level of 5α-R2 in rat bladder epithelial cells (Figure 5). The 5α-R1 is distributed in androgen-independent tissues such as liver and skin, and 5α-R2 is distributed in androgen-dependent tissues [35,36]. Therefore, we hypothesized that ergosterol reduces the expression level of 5α-R2, which might contribute to the suppression of androgen signaling. In addition, ergosterol was found to decrease AR expression in bladder epithelial cells (Figure 5). Therefore, ergosterol reduces the metabolic conversion of testosterone to DHT by decreasing the expression level of 5α-R2 in the bladder and decreases the expression of AR in bladder epithelial cells, thereby weakening androgen signaling. This finding suggests that the carcinogenesis is suppressed. It has been reported that the expression level of AR is upregulated by DHT [37]. Therefore, it is thought that the key to the suppression of bladder carcinogenesis due to ergosterol is the decrease in the expression of 5α-reductase, but there are many unclear points regarding its regulation mechanism. By investigating the DHT concentration in the bladder cells, we believe that the involvement of 5α-reductase in the suppressive action of bladder carcinogenesis by ergosterol will be clarified.

The results of this study clarified that ergosterol inhibits bladder carcinogenesis by modulating various aspects of the cell cycle, inflammation-related signaling, and androgen signaling. Reports have indicated that (1) high expression of cyclin D1 after TUR treatment shortens the time to recurrence in humans [38], (2) COX inhibitors may have a suppressive effect on bladder cancer [39], and (3) patients treated with antiandrogen therapy have a low rate of bladder cancer recurrence [40,41]. Although the prognosis of bladder cancer is relatively better than that of other carcinomas, the recurrence rate is very high. That is, bladder cancer repeatedly recurs, and when the disease progresses from superficial cancer to invasive cancer, the prognosis is poor. Therefore, preventing recurrence is an important issue. The action of ergosterol demonstrated in this study plays a very important role in preventing bladder cancer recurrence. Future clinical application of this preventive effect is expected.

## Figures and Tables

**Table 1:** Primer sequences used for real-time PCR.

Gene	Forward	Reverse
Cyclin D1	CCAGCCGCAATGCTGTAG	TTGGGACGCCTCAGCTAAG
COX-1	AAGGAGATGGCCGCTGAGTT	AGGAGCCCCCATCTCTATCA
COX-2	GCTGATGACTGCCCAACTC	GATCCGGGATGAACTCTCTC
5α-R1	GCTGTACGAGTACATTCGTC	CCCTGATCAGAACCGGGAA
5α-R2	GGGAGCTCTAACCCAATTTC	CCTCTTCAGATCATACCGTG
AR	CCCTCCCATGGCACATTTTG	TTGGTTGGCACACAGCACAG
18S rRNA	GTCTGTGATGCCCTTAGATG	AGCTTATGACCCGCACTTAC

**Table 2:** Inhibitory effect of ergosterol on bladder carcinogenesis.

Group	Number of Con A-Dependent Aggregates	Inhibition Rate (%)
Control	1 ± 1	-
Carcinogenesis	11 ± 3 *	-
Ergosterol	2 ± 1 #	90

The bladders were removed from rats in the control, carcinogenesis, and ergosterol-treated groups. Agglutination of urinary bladder cells in each group was induced by Concanavalin A (Con A), with or without α-methylmannoside (α-MM). Three assays, each on a pooled cell suspension from 2 rats, were carried out in each group of six rats. The data are presented as the means ± SDs. Tukey’s test, * *p* < 0.05 vs. the control group, # *p* < 0.05 vs. the carcinogenesis group.
